# Genospecies diversity of Lyme disease spirochetes in rodent reservoirs.

**DOI:** 10.3201/eid0502.990218

**Published:** 1999

**Authors:** D Richter, S Endepols, A Ohlenbusch, H Eiffert, A Spielman, F R Matuschka

**Affiliations:** Medizinische Fakultät der Humboldt-Universität zu Berlin, Germany. dania.richter@charite.de

## Abstract

To determine whether particular Borrelia burgdorferi s.l. genospecies associate solely with rodent reservoir hosts, we compared the genospecies prevalence in questing nymphal Ixodes ticks with that in xenodiagnostic ticks that had fed as larvae on rodents captured in the same site. No genospecies was more prevalent in rodent-fed ticks than in questing ticks. The three main spirochete genospecies, therefore, share common rodent hosts.


### Genospecies Diversity of Lyme Disease Spirochetes in Rodent Reservoirs

**Dania Richter,*§ Stefan Endepols,† Andreas Ohlenbusch,‡ Helmut Eiffert,‡ Andrew Spielman,§ and Franz-Rainer Matuschka*§**
*Medizinische Fakultät der Humboldt-Universität zu Berlin, Berlin, Germany; †Geschäftsbereich Tiergesundheit, Bayer AG, Leverkusen, Germany; ‡Universitätskliniken der Georg-August-Universität Göttingen, Göttingen, Germany; and §Harvard School of Public Health, Boston, Massachusetts, USA

To determine whether particular *Borrelia burgdorferi* s.l. genospecies associate solely with rodent reservoir hosts, we compared the genospecies prevalence in questing nymphal *Ixodes* ticks with that in xenodiagnostic ticks that had fed as larvae on rodents captured in the same site. No genospecies was more prevalent in rodent-fed ticks than in questing ticks. The three main spirochete genospecies, therefore, share common rodent hosts.

The several genospecies of the Lyme disease spirochete, *Borrelia burgdorferi* sensu lato, that infect people in Eurasia produce a broad spectrum of human disease. Particular genospecies have been associated with characteristic symptoms; chronic skin disease, for example, results from infection by *Borrelia afzelii* (1). Rodents (e.g., various *Apodemus* mice [2], Norway rats, *Rattus norvegicus* [3,4], edible dormice, *Glis glis* [5]) serve unambiguously as reservoir hosts for Lyme disease spirochetes. Although each major European genospecies has been associated with birds (6), *B. afzelii* is thought to perpetuate in rodents and *B. garinii* is thought to perpetuate in avian reservoir hosts (7,8). The European vector of Lyme disease, *Ixodes ricinus*, maintains an unusual diversity of pathogens in an extraordinarily broad array of hosts.

The vertebrates that are infested most frequently by arthropods generally also serve as reservoir hosts for any pathogens transmitted by these vectors. The transmission cycle would likely be broken if some vector acquired a pathogen from one host but injected it into an ill-adapted host. If *B. afzelii* thrived mainly in rodents (7), transmission might not be sustained if a larval tick acquired these spirochetes from a mouse and attached subsequently as a nymph to a bird. If *B. garinii*, on the other hand, thrived mainly in birds, a corresponding diversion of its vector to a rodent would similarly result in transmission failure. Efficient perpetuation of rodent-borne *B. afzelii* as well as bird-borne *B. garinii* by the same subadult *I. ricinus* vector ticks, therefore, would seem paradoxical.

Because rodents may serve as reservoir hosts for both *B. afzelii* and *B. garinii*, we determined whether the genospecies distribution of spirochetes naturally infecting rodents corresponds to that in questing vector ticks. We compared the genospecies diversity of spirochetes infecting questing nymphal *I. ricinus* ticks with that of spirochetes infecting nymphs that had fed as larvae on Norway rats or on yellow-necked mice, *Apodemus flavicollis*, captured in the same site.

### The Study

Norway rats and yellow-necked mice were captured in an urban park in Magdeburg, Germany, from June through September 1994, by using apple- and bread-baited Tomahawk traps (Tomahawk Live Trap Company, Tomahawk, WI) and apple- and rodent chow–baited Longworth traps (Longworth Scientific Instruments, Abingdon, UK) (4). Each captured rodent was caged over water for 1 week to permit detachment of all ticks that had attached to these rodents in the field. Subsequently, rodents were

Address for correspondence: Dania Richter, Institut für Pathologie, Charité, Medizinische Fakultät der Humboldt-Universität zu Berlin, Malteserstraße 74-100, 12249 Berlin, Germany; fax: 49-30-776-2085; e-mail: dania.richter@charite.de.

infested with noninfected laboratory-bred *I. ricinus* larvae for xenodiagnosis. To confirm that larvae used for xenodiagnosis were free of spirochetes, a sample of each batch was routinely fed on a laboratory-bred mouse; no spirochetes were found when midguts of 20 of the resulting nymphs were examined by dark-field microscopy, and subsequent xenodiagnosis of the mouse showed no evidence of spirochetes. The water was changed and inspected at least twice a day and detached larvae were removed promptly. Engorged larvae were enclosed in screened vials and kept at 20±2°C in sealed desiccator jars over supersaturated $MgSO_4$ under a light-dark regimen (16:8) until they molted.

Questing *I. ricinus* ticks were collected from the vegetation in the study site by using flannel flags. Collected ticks were confined in screened vials and stored at 15±1°C in sealed desiccator jars containing supersaturated $MgSO_4$ until they were examined microscopically, identified to stage, and prepared for polymerase chain reaction (PCR) analysis.

### DNA Extraction, Amplification, and Sequencing

Total DNA from the ear pinnae of field-caught rodents was prepared with a QiAamp Tissue kit (Qiagen GmbH, Hilden, Germany). Engorged *I. ricinus* larvae that served for xenodiagnosis were permitted to molt to nymphs. The opisthosoma was then opened, andTthe contained mass of soft tissue was dissected out into 400 µl ice-cold Tris-EDTA buffer (pH 7.4, 10mM Tris, 1mM EDTA). Suspensions of tick tissue were adjusted to 0.5% sodium dodecyl sulfate, 0.2M NaCl, 10mM Tris, and 5mM EDTA at pH 8.0; proteinase K (Boehringer Mannheim, Mannheim, Germany) was added (0.2mg/ml) and incubated at 56°C for 3 hours. DNA was extracted with phenol-chloroform. Ethanol-precipitated DNA was resuspended in 50 µl distilled water.

*Borrelia* genospecies were characterized by amplifying and sequencing a 400-nucleotide segment of the gene encoding the outer surface protein A (OspA) (9-11). To increase the sensitivity for detection of spirochetal DNA in ticks, we used nested PCR (10). Aliquots of DNA suspensions (20 µl) were diluted to 50 µl by using 200 µM each of deoxynucleoside triphosphate, 4mM $MgCl_2$, 10mM Tris at pH 8.3, 50mM KCl, 0.01% Tween-20, 0.01% gelatin, and 0.8 units Taq polymerase (Amersham, Braunschweig, Germany), as well as 10pmol of the outer primer pair or 20pmol of the inner primer pair. We used the following primer sequences (5'-3') of the *ospA* gene (9): outer primers GGTCTAATATTAGCCTT AATAGGCATG (positions 169-194) and TCAG CAGCTAGAGTTCCTTCAAG (positions 665-643); inner primers CATGTAAGCAAAATGTTAG CAGCC (positions 191-214) and CTGTGTATTCA AGTCTGGTTCC (positions 589-568). The mixture was overlaid with mineral oil (Sigma, Deisenhofen, Germany), placed in a thermocycler (Omnitech, Heidelberg, Germany), heated for 2 min at 95°C, and subjected to 40 cycles of 20 sec denaturation at 95°C, 20 sec each for the first annealing reaction at 59°C, for the second at 61°C and with a 20-sec extension at 72°C. After the first amplification with the outer set of primers, 5 µl of product was transferred to a fresh tube containing 45 µl of reaction mixture as described for the inner set of primers. PCR products were detected by electrophoresis in a 1.5% agarose gel stained with ethidium bromide. DNA was extracted, reaction vials were prepared for amplification, and products were electrophoresed in separate rooms. For comparison, each series of PCR amplification included two laboratory-reared nymphs that had fed as larvae on *B. afzelii*-infected jirds (*Meriones unguiculatus*) and two that had fed on noninfected jirds.

Each PCR amplification product was purified by using a QIAquick-Spin PCR column (Qiagen). Amplified DNA fragments were directly sequenced in both directions using the inner primers by the dideoxynucleotide chain-termination method on an ABI 373 DNA-sequencer according to the manufacturer's instructions (Applied Biosystems, Foster City, CA). Each resulting sequence was compared with sequences of the same fragment representing *B. burgdorferi* sensu stricto, *B. afzelii,* and five serotypes of *B. garinii* (9-11); an exact fit was required. With this technique, each of these genospecies can be detected with equal sensitivity; the technique detects and identifies two different coinfecting spirochete genospecies, even when one is five times as numerous as the other (10).

### The Findings

We first described the frequency of spirochetal infection and the distribution of spirochete genospecies infecting questing *I. ricinus* ticks collected from vegetation. Although all three spirochete genospecies were present in these ticks, *B. garinii* was somewhat more prevalent than *B. afzelii*; *B. burgdorferi* s.s. was infrequent (Table 1). A similar distribution of genospecies was found in questing adult ticks (data not shown). Spirochetes of each of the major pathogenic European genospecies infect questing vector ticks at our study site.

We then identified the spirochete genospecies that naturally infected rodents transmitted to xenodiagnostic larvae. Spirochetes were present in virtually all nymphal ticks that had fed as larvae on rats captured at the study site and in approximately half of those that had fed on mice (Table 1). Each of the three main genospecies was present. The 1:2:1 ratio of *B. afzelii* to *B. garinii* to *B. burgdorferi* s.s. in ticks that fed on rats did not differ from the 3:3:1 ratio in nymphal ticks that fed on mice (Chi-square, p=0.7). The 2:3:1 overall ratio of genospecies in xenodiagnostic ticks did not differ from the 4:5:1 ratio in questing ticks (Chi-square, p=0.9). In contrast, only *B. afzelii* DNA was amplified from the ear pinnae of these hosts (data not shown). The array of spirochete genospecies acquired by ticks feeding on field-derived rodents is similar to that in questing ticks but differs sharply from that present in skin samples of these rodents.

To examine the transmissibility of the three genospecies of Lyme disease spirochetes, we compared genospecies diversity in *I. ricinus* ticks that had fed on individual rodents. At least two such infected ticks were available for each of these rats and for all but one mouse; that mouse was excluded from this analysis (Table 2). Although more than one spirochete genospecies infected the cohort of ticks that fed on each rodent, no individual tick was infected with more than one genospecies. The *B. garinii* genospecies infected somewhat more ticks than did *B. afzelii*, and *B. afzelii* infected somewhat more than did *B. burgdorferi* s.s. We found that no particular spirochete genospecies is transmitted more frequently than any other.

Table 1. *Borrelia* genospecies diversity in questing nymphal *Ixodes ricinus* ticks and in nymphal ticks that had fed as larvae on Norway rats, *Rattus norvegicus*, or yellow-necked mice, *Apodemus flavicollis*

| | *Borrelia* prevalence | | Distribution of genospecies | | | |
|---|---|---|---|---|---|---|
| | | | No. | % with *Borrelia* | | |
| Tick source | No. tested | % infected | ticks tested | *afz*[a] | *gar* | *bur* |
| Questing | 112 | 9 | 10 | 40 | 50 | 10 |
| Fed on rat[b] | 50 | 86 | 25 | 28 | 52 | 20 |
| Fed on mouse[c] | 36 | 42 | 15 | 40 | 47 | 13 |

[a]*afz,afzelii*; *gar, garinii*; *bur*, *burgdorferi* sensu stricto.
[b]Ticks fed on five infected rats.
[c]Ticks fed on four infected mice.

Table 2. Natural infectivity for xenodiagnostic *Ixodes ricinus* ticks of various genospecies of Lyme disease spirochetes

| | | No. ticks infected by *Borrelia* | | |
|---|---|---|---|---|
| Infected rodent | Rodent no. | *afzelii* | *garinii* | *burgdorferi* sensu stricto |
| *Rattus norvegicus* (Norway rats) | 1 | 2 | 2 | 1 |
| | 2 | 0 | 3 | 2 |
| | 3 | 1 | 4 | 0 |
| | 4 | 3 | 2 | 0 |
| | 5 | 1 | 2 | 2 |
| | Total | 7 | 13 | 5 |
| *Apodemus flavicollis* (Yellow-necked mice) | 1 | 3 | 3 | 1 |
| | 2 | 3 | 2 | 0 |
| | 3 | 0 | 1 | 1 |
| | Total | 6 | 6 | 2 |

### Conclusions

Although *B. garinii* spirochetes most frequently infect questing ticks in our German study site, the other two major genospecies predominate elsewhere in Europe. *B. afzelii* predominates in questing ticks in four European sites (12-14) and *B. burgdorferi* s.s. in four others (Table 3) (7,15-17). In three sites, different combinations of two genospecies predominate (7,15,18). No regional pattern of genospecies diversity in questing ticks seems evident, nor does the sampling method influence the genospecies ratio. If *B. garinii* were to perpetuate in an avian reservoir (8,19) and *B. afzelii* in rodents (7), the relative availability of these hosts to vector ticks would determine genospecies distribution in a site. Alternatively, the founder's principle (i.e., in the absence of some selective force, the present ratio randomly reflects that of the past) may influence genospecies distribution. No available longitudinal study, however, permits such a temporal

Table 3. *Borrelia* genospecies diversity in questing *Ixodes ricinus* ticks from various European sites

| Site[a] | | No. infected ticks | *Borrelia* genospecies Relative ratio *afz*[b] | *gar* | *bur* | % mixed | Sampling method | Ref. |
|---|---|---|---|---|---|---|---|---|
| CH | 1 | 7 | 1 | 2 | 2 | 8 | Culture | 7 |
| | 2 | 6 | 0 | 1 | 5 | 0 | Culture | 7 |
| | v | 50 | 1 | 6 | 9 | 0 | Culture | 17 |
| CR | v | 56 | 19 | 3 | 1 | 20 | Direct | 13 |
| D | 1 | 52 | 5 | 11 | 1 | 2 | Direct | 1 |
| | 2 | 10 | 4 | 5 | 1 | 0 | Direct | TA[c] |
| F | 1 | 25 | 10 | 5 | 1 | 24 | Direct | 12 |
| GB | 1 | 16 | 0 | 10 | 1 | 0 | Direct | 8 |
| IR | 1 | 11 | 1 | 4 | 5 | 9 | Direct | 15 |
| | 2 | 10 | 0 | 2 | 1 | 0 | Direct | 15 |
| | 3 | 20 | 4 | 1 | 4 | 15 | Direct | 15 |
| NL | 1 | 15 | 1 | 1 | 0 | 14 | Direct | 18 |
| | v | 63 | 1 | 9 | 14 | 0 | Culture | 16 |
| SL | 1 | 47 | 3 | 2 | 1 | 5 | Culture | 14 |
| | v | 13 | 12 | 1 | 0 | 0 | Culture | 14 |

[a]CH, Switzerland; CR, Croatia; D, Germany; F, France; GB, Great Britain; IR, Ireland; NL, Netherlands; SL, Slovenia; v, sum of various sites in a country.
[b]*afz, Borrelia afzelii*; *gar, Borrelia garinii*; *bur, Borrelia burgdorferi* sensu stricto.
[c]This article.

interpretation. The distribution of European *B. burgdorferi* s.l. genospecies appears to be site-specific and may be random. For this reason, genospecies comparisons designed to differentiate reservoirs of these spirochetes should be based on diversity within a single site.

The method used to sample spirochetes from a mammal host may bias the results in favor of one or another genospecies. When spirochetes are isolated from European mice by culturing segments of their ear pinnae, the *B. afzelii* genospecies seems to predominate (7). In direct identification by PCR and sequence analysis, we confirmed the sole presence of this genospecies in ear pinnae from Norway rats and yellow-necked mice captured at our study site. A more diverse array of genospecies, however, infects ticks questing at this site. Although this finding would suggest that *B. afzelii* is the sole spirochete genospecies infecting these rodents, xenodiagnostic observations indicate that samples based on ear biopsies do not reflect the total diversity of spirochetes infecting such a rodent. Interestingly, *B. burgdorferi* s.s. is readily detected in earpunch samples taken from American mice (20). The apparent association of *B. afzelii* with European rodent hosts may derive from a sampling artifact.

In contrast to the reported association of *B. afzelii* with rodents, *B. garinii* is reported to depend on avian reservoir hosts (7,8,21). Evidence for such a genospecies-specific avian reservoir derives originally from observations on an isolated island site where numerous seabirds nested and where no other spirochetes were detected (19). DNA characteristic of *B. garinii* was detected in questing and seabird-feeding *I. uriae* ticks and in the footweb of a razorbill. This genospecies also predominates in pheasants in a British site in which no *B. afzelii* spirochetes are evident (8). Although *B. garinii* infects bird-feeding *I. persulcatus* ticks in a Japanese site and *B. afzelii* infects those feeding on rodents (21), another Japanese study detected both genospecies in voles (22). Experimental studies, however, suggest that birds are relatively incompetent as hosts of Lyme disease spirochetes. Domestic chickens, for example, become only transiently competent (23), and European blackbirds, *Turdus merula*, and Canary finches, *Serinus canarius*, appear not to become infected (3,24). Spirochetes (probably *B. burgdorferi* s.s.), however, have been detected in larval *I. dammini* taken from particular North American birds (25). Evidence of nonspecificity in avian hosts is provided by observations in Scandinavia, where spirochetes of the three major genospecies infect larval ticks that had fed on various passerine birds (6). Indeed, our finding that vector ticks ingest *B. garinii* spirochetes from rodents at least as frequently as *B. afzelii* argues against the concept of genospecies-reservoir specificity.

Surprisingly, few ticks questing in Europe appear to contain more than one spirochete genospecies (Table 3). No more than one in four ticks is multiply infected (12) (generally far fewer). None of the questing ticks collected in our European study site appear to contain more than one kind of spirochete, nor did any laboratory-reared ticks permitted to feed on rodents captured in this site. These rodents, however, were multiply infected: each rodent infected some of the ticks that fed on them with two or more kinds of spirochetes. Although our diagnostic procedure may more reliably detect DNA of the more abundant of two genospecies coinfecting a tick, our findings may have a biologic basis. We suggest that a single genospecies becomes established in a tick far more frequently than do multiple genospecies.

Host specificity of a vector contributes powerfully to the intensity of transmission of a tick-borne pathogen. Such a pathogen would fail to perpetuate unless the host-range of its vector corresponds closely to that of its reservoir. It would seem paradoxical if the same *I. ricinus* population maintained spirochetes of one genospecies in birds, while maintaining another in rodents. This "one-vector-one-reservoir" principle is consistent with our discovery of a similar genospecies distribution in rodents and in ticks questing at our study site. If the *B. garinii* genospecies were to predominate there in birds (7), such spirochetes would have been more prevalent in questing ticks than in ticks that had engorged on sympatric rodents; however, this is not the case. Our site-specific observation that the genospecies distribution in rodent-fed ticks reflects that in questing ticks argues that all three pathogenic spirochete genospecies share common reservoir hosts.

### Acknowledgments

This study was supported by grants Ma 942/7-1 and Ma 942/10-1 from the Deutsche Forschungsgemeinschaft. Dr. Richter was supported by a stipend "Infektionsforschung" from the Bundesministerium für Forschung und Technik.

Dr. Richter is a postdoctoral fellow conducting joint research in the laboratories of Dr. Matuschka, Charité Medical School, Humboldt Universität zu Berlin, and Dr. Spielman at the Harvard School of Public Health. Her research interests focus on the immunologic and molecular interface of the host-vector-pathogen relationship in the epizootiology of Lyme disease.

### References

1. Ohlenbusch A, Matuschka F-R, Richter D, Christen H-J, Thomssen R, Spielman A, et al. Etiology of the *acrodermatitis chronica atrophicans* lesion in Lyme disease. J Infect Dis 1996;174:421-3.
2. Matuschka F-R, Fischer P, Heiler M, Richter D, Spielman A. Capacity of European animals as reservoir hosts for the Lyme disease spirochete. J Infect Dis 1992;165:479-83.
3. Matuschka F-R, Eiffert H, Ohlenbusch A, Richter D, Schein E, Spielman A. Transmission of the agent of Lyme disease on a subtropical island. Tropical Medicine and Parasitology 1994;45:39-44.
4. Matuschka F-R, Endepols S, Richter D, Ohlenbusch A, Eiffert H, Spielman A. Risk of urban Lyme disease enhanced by the presence of rats. J Infect Dis 1996;174:1108-11.
5. Matuschka F-R, Eiffert H, Ohlenbusch A, Spielman A. Amplifying role of edible dormice in Lyme disease transmission in Central Europe. J Infect Dis 1994;170:122-7.
6. Olsen B, Jaenson TGT, Bergström S. Prevalence of *Borrelia burgdorferi* sensu lato-infected ticks on migrating birds. Appl Environ Microbiol 1995;61:3082-7.
7. Humair P-F, Peter O, Wallich R, Gern L. Strain variation of Lyme disease spirochetes isolated from *Ixodes ricinus* ticks and rodents collected in two endemic areas in Switzerland. J Med Entomol 1995;32:433-8.
8. Kurtenbach K, Peacey M, Rijpkema SGT, Hoodless AN, Nuttall PA, Randolph SE. Differential transmission of the genospecies of *Borrelia burgdorferi* sensu lato by game birds and small rodents in England. Appl Environ Microbiol 1998;64:1169-74.
9. Eiffert H, Ohlenbusch A, Fehling W, Lotter H, Thomssen R. Nucleotide sequence of the *ospAB* operon of a *Borrelia burgdorferi* strain expressing OspA but not OspB. Infect Immun 1992;60:1864-8.
10. Eiffert H, Ohlenbusch A, Christen H-J, Thomssen R, Spielman A, Matuschka F-R. Nondifferentiation between Lyme disease spirochetes from vector ticks and human cerebrospinal fluid. J Infect Dis 1995;171:476-9.
11. Ohlenbusch A. Beiträge zur Diagnostik und Pathogenese der Lyme-Borreliose und zur Transmission des Erregers *Borrelia burgdorferi* [dissertation]. Göttingen (Germany): Georg-August-Universität; 1996.
12. Pichon B, Godfroid E, Hoyois B, Bollen A, Rodhain F, Perez-Eid C. Simultaneous infection of *Ixodes ricinus* nymphs by two *Borrelia burgdorferi* sensu lato species: possible implications for clinical manifestations. Emerg Infect Dis 1995;1:89-90.
13. Rijpkema S, Golubic D, Molkenboer M, Verbeek-De Kruif N, Schellekens J. Identification of four genomic groups of *Borrelia burgdorferi* sensu lato in *Ixodes ricinus* ticks collected in a Lyme borreliosis endemic region in northern Croatia. Exp Appl Acarol 1996;20:23-30.
14. Strle F, Cheng Y, Nelson JA, Picken MM, Bouseman JK, Picken RN. Infection rate of *Ixodes ricinus* ticks with *Borrelia afzelii*, *Borrelia garinii*, and *Borrelia burgdorferi* sensu stricto in Slovenia. Eur J Clin Microbiol Infect Dis 1995;14:994-1001.
15. Kirstein F, Rijpkema S, Molkenboer M, Gray JS. Local variations in the distribution and prevalence of *Borrelia burgdorferi* sensu lato genomospecies in *Ixodes ricinus* ticks. Appl Environ Microbiol 1997;63:1102-6.
16. Nohlmans LKME, De Boer R, Van den Bogard AEJM, Van Boven CPA. Genotypic and phenotypic analysis of *Borrelia burgdorferi* isolates from the Netherlands. J Clin Microbiol 1995;33:119-25.
17. Peter O, Bretz A-G, Bee D. Occurrence of different genospecies of *Borrelia burgdorferi* sensu lato in ixodid ticks in Valais, Switzerland. Eur J Epidemiol 1995;11:463-7.
18. Rijpkema SGT, Molkenboer MJCH, Schouls LM, Jongejan F, Schellekens JFP. Simultaneous detection and genotyping of three genomic groups of *Borrelia burgdorferi* sensu lato in Dutch *Ixodes ricinus* ticks by characterization of the amplified intergenic spacer region between 5S and 23S rRNA genes. J Clin Microbiol 1995;33:3091-5.
19. Olsen B, Jaenson TGT, Noppa L, Bunikis J, Bergström S. A Lyme borreliosis cycle in seabirds and *Ixodes uriae* ticks. Nature 1993;362:340-2.

20. Sinsky RJ, Piesman J. Ear punch biopsy method for detection and isolation of *Borrelia burgdorferi* from rodents. J Clin Microbiol 1989;27:1723-7.
21. Nakao M, Miyamoto K, Fukunaga M. Lyme disease spirochetes in Japan: enzootic transmission cycles in birds, rodents, and *Ixodes persulcatus* ticks. J Infect Dis 1994:170:878-82.
22. Ishiguro F, Takada N, Nakata K. Reservoir competence of the vole, *Clethrionomys rufocanus* bedfordiae, for *Borrelia garinii* or *Borrelia afzelii*. Microbiol Immunol 1996;40:67-9.
23. Piesman J, Dolan MC, Schriefer ME, Burkot TR. Ability of experimentally infected chickens to infect ticks with the Lyme disease spirochete, *Borrelia burgdorferi*. Am J Trop Med Hyg 1996;54:294-8.
24. Matuschka F-R, Spielman A. Loss of Lyme disease spirochetes from *Ixodes ricinus* ticks feeding on European blackbirds. Exp Parasitol 1992;74:151-8.
25. Rand PW, Lacombe EH, Smith RP, Ficker J. Participation of birds in the emergence of Lyme disease in southern Maine. J Med Entomol 1998;35:270-6.
